# Estimating numbers of intracellular molecules through analysing fluctuations in photobleaching

**DOI:** 10.1038/s41598-019-50921-7

**Published:** 2019-10-23

**Authors:** Elco Bakker, Peter S. Swain

**Affiliations:** School of Biological Sciences, University of Edinburgh, Edinburgh, United Kingdom

**Keywords:** Biological fluorescence, Single-cell imaging, Time series

## Abstract

The impact of fluorescence microscopy has been limited by the difficulties of expressing measurements of fluorescent proteins in numbers of molecules. Absolute numbers enable the integration of results from different laboratories, empower mathematical modelling, and are the bedrock for a quantitative, predictive biology. Here we propose an estimator to infer numbers of molecules from fluctuations in the photobleaching of proteins tagged with Green Fluorescent Protein. Performing experiments in budding yeast, we show that our estimates of numbers agree, within an order of magnitude, with published biochemical measurements, for all six proteins tested. The experiments we require are straightforward and use only a wide-field fluorescence microscope. As such, our approach has the potential to become standard for those practising quantitative fluorescence microscopy.

## Introduction

In fluorescence microscopy, converting measurements of fluorescence into numbers of molecules is a long-standing challenge^1^. This deficit limits our ability both to combine fluorescence measurements from different experiments and to apply quantitative analyses of time series that often must assume known numbers of proteins^2–4^.

Although fluorescence standards^1^ and the collection of techniques known as fluorescence fluctuation spectroscopy – the most well known of which are fluorescence correlation spectroscopy (FCS)^5,6^ and analysis of photon-counting histograms (PCH)^7^ – provide a solution, neither are yet commonly adopted to calibrate, for example, time-lapse imaging in cell and systems biology^8^.

Instead, fluctuation-based methods have been developed, such as those that measure fluctuations in the distribution of fluorescent proteins between daughter cells at cell division^9–11^. These approaches, however, have been applied mostly to bacteria, are unsuitable for non-dividing cells^12^, and do not straightforwardly extend to species that exhibit differences in size between mothers and daughters.

A second approach is to study fluctuations in stochastic processes of decay. Inhibiting translation and transcription has allowed the fluorescence per molecule to be estimated in mammalian cells^13^, but the stability of fluorescent proteins can make these experiments time consuming. An alternative technique is to deliberately induce photobleaching^14^: the process by which fluorophores cease to fluoresce when continuously excited. This method has been applied *in vitro*^15,16^ and to bacteria^17^, but the analysis relies on photobleaching exhibiting an exponential decay^14^, which is expected for single molecules but not necessarily for the fluorescence of cells^18^.

Here we develop a method for estimating numbers from deliberately photo-bleached cells that works on the wide-field microscopes used for time-lapse imaging and requires no specialized equipment. We verify our approach using six different proteins tagged with Green Fluorescent Protein (GFP) in budding yeast. In all cases, our estimates of the numbers of molecules are within an order of magnitude of estimates made using biochemical techniques, such as quantitative Western blotting and mass spectrometry.

**Figure 1:**
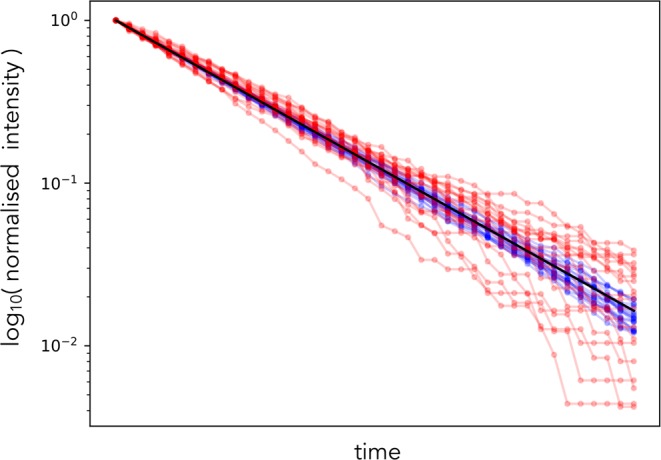
The magnitude of fluctuations is determined by numbers of molecules, which we illustrate by simulating stochastic exponential decay for both a small number of molecules in red and a large number in blue. By normalizing to the same starting value, these simulated time series show fluctuations of a different magnitude depending on their initial numbers of molecules. The mean behaviour is common (black line), but the data for low numbers of molecules (red) shows larger deviations from the mean than the data for high numbers of molecules (blue).

Before beginning, we remind the reader that fluctuation analyses work because the magnitude of fluctuations in fluorescence are determined not by the concentration of the fluorescent molecules but by their numbers. A fluorescent measurement, *Y*, is given by the product of the brightness per molecule, *ν*, and the number of fluorescent molecules, *X*, and is therefore agnostic to their individual values. If *X* varies with time, the magnitude of the fluctuations in the underlying biochemical process changing *X*, however, typically scale with the mean^19^: ${\rm{Var}}[X]\propto E[X]$. Therefore the variance in the fluorescence, scales as ${\rm{Var}}[Y]\propto {\nu }^{2}E[X]=\nu E[Y]$ because $Y=\nu X$. For a given fluorescence (a given $E[Y]$), large fluctuations (large ${\rm{Var}}[Y]$) therefore imply a high value of *ν* and low numbers of molecules, and vice versa (Fig. 1).

## Results

### Photobleaching *in vivo* has more than one time scale

**Figure 2:**
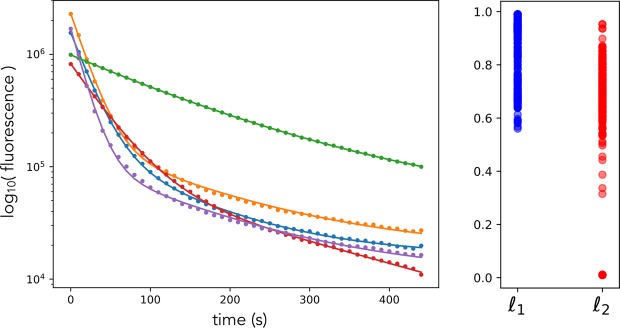
Photobleaching *in vivo* is not described by a homogeneous, single exponential decay. Left: The logarithm of the fluorescence of 5 cells of budding yeast expressing Pgk1-GFP and undergoing photobleaching do not all fall on a straight line as a function of time (c.f. Fig. 1). The data are shown as dots and the fits for double exponential decay as lines, which is modelled for fluorescence at time point *i* as ${f}_{i}={a}_{0}+{a}_{1}{\ell }_{1}^{i}+{a}_{2}{\ell }_{2}^{i}$ with constants *a* and ${\ell }_{1}={{\rm{e}}}^{-{\lambda }_{1}\Delta t}$ and ${\ell }_{2}={{\rm{e}}}^{-{\lambda }_{2}\Delta t}$ for time scales *λ* and time Δ*t* between measurements (here 10 s). All data have been corrected for autofluorescence using wild-type cells that do not express GFP. Right: bleaching is heterogeneous across the population of cells. There is substantial cell-to-cell variation in the best-fit values for ${\ell }_{1}$ and ${\ell }_{2}$.

We obtained time series of photobleaching using budding yeast and GFP-tagged proteins^20^. Cells were fixed and photobleached in sustained illumination with fluorescence measurements taken every 10 seconds (Fig. 2 and Methods). We use fixed cells to remove complications arising from cells synthesising and degrading proteins and from fluorescent proteins maturing during the 6 minutes or so of bleaching.

Our data is incompatible with earlier fluctuation-based methodologies (Methods) because it is not well described by a single exponential decay even after being corrected for autofluorescence (Fig. 2; left). First, the data for multiple cells shows systematic deviations from a single exponential and is better fit by a bi-exponential decay. Second, the decay rates of these two exponentials vary substantially between cells (Fig. 2; right). Multi-exponential photobleaching is common^18^ and can be caused by, for example, differing intracellular micro-environments^21^, molecular rotation^22^, and higher order interactions between excited fluorophores^23^. These phenomena can also cause heterogeneity in parameters between cells.

### A Bayesian approach systematically underestimates

We wish to infer from our data, *ν*, the factor that converts from fluorescence units to absolute numbers^10^. We follow an established Bayesian methodology for stochastic, chemical systems^24^, which uses the linear noise approximation^19^ to describe the dynamics of bleaching and a Kalman filter for the inference. Our model is general: each cell contains two pools of fluorescent molecules that bleach with their own time scale and measurement noise has a normal distribution but with a variance that is up to a quadratic function of the total number of fluorescent molecules (Methods). Using a Markov chain Monte Carlo scheme to sample the posterior distribution, the numbers of molecules we infer are, however, too small (Methods).

We conclude that our model of either bleaching or measurement noise is incorrect or that both are incorrect. Shortcomings in the bleaching model, for example, will generate discrepancies between the model’s behaviour and the cells’ actual behaviour that are correlated over time. We make the common assumption that the measurement noise at each time point is independent, and so the algorithm will interpret any correlated error as coming from bleaching. The magnitude of the fluctuations in bleaching will correspondingly be overestimated and the number of molecules underestimated.

### An estimator for the number of molecules

We therefore propose instead an estimator that is insensitive to the details of the photobleaching. We motivate the estimator by first considering bleaching with a single rate constant, *λ*:

1 $$X\mathop{\to }\limits^{\lambda }{X}_{{\rm{bleached}}}$$

Using the linear noise approximation^19^ and writing $\ell ={{\rm{e}}}^{-\lambda t}$ for the probability of a fluorophore remaining unbleached at the time *t* of interest, we can show (Methods) that from *x*_0_ molecules at $t=0$

2 $${\rm{E}}[{X}_{t}]={x}_{0}\ell $$

and that

3 $${\rm{Var}}[{X}_{t}]={x}_{0}(1-\ell )\ell .$$

Dividing Eq. 3 by ${x}_{0}^{2}$ implies that the magnitude of these normalized fluctuations in ${X}_{t}/{x}_{0}$ scale as $1/{x}_{0}$ (Fig. 1).

Using Eq. 2, we can replace $\ell$ in Eq. 3,

4 $${\rm{Var}}[{X}_{t}]=\frac{{\rm{E}}[{X}_{t}]}{{x}_{0}}({x}_{0}-{\rm{E}}[{X}_{t}])$$

which holds for all *t*. If we ignore measurement noise then fluorescence $Y=\nu X$, and multiplying Eq. 4 by *ν*^2^, assumed to be the same for each molecule, gives

5 $${\rm{Var}}[{Y}_{t}]=\frac{{\rm{E}}[{Y}_{t}]}{{x}_{0}}({y}_{0}-{\rm{E}}[{Y}_{t}])$$

and so our estimator for *x*_0_ is

6 $${\hat{x}}_{0}=\frac{{\rm{E}}[{Y}_{t}]({y}_{0}-{\rm{E}}[{Y}_{t}])}{{\rm{Var}}[{Y}_{t}]}$$

for any time $t > 0$. Given *y*_0_, ${\rm{E}}[{Y}_{t}]$, ${\rm{Var}}[{Y}_{t}]$, and no measurement noise, we can show that ${\hat{x}}_{0}$ is a maximum likelihood estimator of *x*_0_ (Methods).

To gain intuition about when the estimator should be reliable, consider the extreme case where each molecules has its own brightness, *ν*_*i*_, and bleaches with its own rate. This bleaching should be independent and if ${\ell }_{i}$ is the probability of molecule *i* remaining unbleached at a time *t*, then

7 $$\begin{array}{rcl}{\rm{E}}[{Y}_{t}] & = & \mathop{\sum }\limits_{i=1}^{{x}_{0}}\,{\nu }_{i}{\ell }_{i}\\ {\rm{Var}}[{Y}_{t}] & = & \mathop{\sum }\limits_{i=1}^{{x}_{0}}\,{\nu }_{i}^{2}{\ell }_{i}(1-{\ell }_{i})\\ {y}_{0} & = & \mathop{\sum }\limits_{i=1}^{{x}_{0}}\,{\nu }_{i}\end{array}$$

from Eqs 2 and 3. Letting measurement noise have variance, ${\sigma }_{e}^{2}$, then the measured variance ${\rm{Var}}[{Y}_{t}]$ will be increased by ${\sigma }_{e}^{2}$ above the value in Eqs 7, and 6 becomes

8 $${\hat{x}}_{0}=\frac{\sum _{i}\,{\nu }_{i}{\ell }_{i}\,\sum _{j}\,({\nu }_{j}-{\nu }_{j}{\ell }_{j})}{{\sigma }_{e}^{2}+\sum _{i}\,{\nu }_{i}^{2}{\ell }_{i}(1-{\ell }_{i})}.$$

The *ν*_*i*_ and ${\ell }_{i}$ for each molecule are unknown, and to proceed we assume that they are samples from a probability distribution, $P(\nu ,\ell )$. Then ${\sum }_{i}\,{\nu }_{i}{\ell }_{i}$ is an empirical estimate for ${x}_{0}{\rm{E}}[\nu \ell ]$ because there are *x*_0_ terms in the sum. We can write Eq. 8 as

9 $${\hat{x}}_{0}=\frac{{x}_{0}}{1+\epsilon }$$

where

10 $$\epsilon =\frac{\frac{{\sigma }_{e}^{2}}{{x}_{0}}+{\rm{Cov}}[\nu ,\nu \ell ]-{\rm{Var}}[\nu \ell ]}{{\rm{E}}[\nu \ell ]({\rm{E}}[\nu ]-{\rm{E}}[\nu \ell ])}$$

after re-arranging. Equations 9 and 10 imply that ${\hat{x}}_{0}$ underestimates *x*_0_ if measurement noise is sufficiently large,

11 $$\frac{{\sigma }_{e}^{2}}{{x}_{0}}+{\rm{Cov}}[\nu ,\nu \ell ] > {\rm{Var}}[\nu \ell ]$$

and overestimates if both measurement noise is sufficiently small and ${\rm{Var}}[\nu \ell ] > {\rm{Cov}}[\nu ,\nu \ell ]$ such that $\epsilon  < 0$.

For the estimates to be accurate, $|\epsilon |\ll 1$, which holds if

12 $$|\frac{{\sigma }_{e}^{2}}{{x}_{0}}+{\rm{C}}{\rm{o}}{\rm{v}}[\nu ,\nu \ell ]-{\rm{V}}{\rm{a}}{\rm{r}}[\nu \ell ]|\ll {\rm{E}}[\nu \ell ]({\rm{E}}[\nu ]-{\rm{E}}[\nu \ell ]).$$

If *σ*_*e*_ is too large, the estimate of *x*_0_ fails.

If *ν* is homogeneous so that $\nu ={\rm{E}}[\nu ]$ for all molecules, then Eq. 12 simplifies to

13 $$\frac{{\sigma }_{e}^{2}}{{\rm{E}}{[\nu ]}^{2}{x}_{0}}\ll {\rm{E}}[\ell ]-{\rm{E}}{[\ell ]}^{2}+{\rm{Var}}[\ell \mathrm{].}$$

We note that ${\rm{E}}[\ell ] > {\rm{E}}[{\ell }^{2}]$ because $0 < \ell  < 1$. Equation 13 is most easily satisfied midway through the bleaching when ${\rm{E}}[\ell ]=1/2$ and the difference between ${\rm{E}}[\ell ]$ and ${\rm{E}}{[\ell ]}^{2}$ is maximum.

If $\ell ={\rm{E}}[\ell ]$ for all molecules, then Eq. 12 simplifies to

14 $$\frac{{\sigma }_{e}^{2}}{{\rm{E}}{[\nu ]}^{2}{x}_{0}}\ll ({\rm{E}}[\ell ]-{\rm{E}}{[\ell ]}^{2})(1-\frac{{\rm{Var}}[\nu ]}{{\rm{E}}{[\nu ]}^{2}})\mathrm{.}$$

Equation 14 implies that Eq. 6 fails if the variation in *ν* is so high that ${\rm{Var}}[\nu ] > E{[\nu ]}^{2}$ and again is most easily satisfied midway through the bleaching.

In summary, our estimator, Eq. 6, can work even in the completely heterogenous case where each molecule has its own brightness and rate of bleaching, but is more sensitive to variation in *ν* than in $\ell$. Measurement noise if sufficiently high will cause underestimation and if too high will, as expected, undermine accuracy.

### Using the estimator

After correcting for autofluorescence, flatfield (inhomogeneous illumination), and background (Methods), the data set comprises *n*_*r*_ cells with a time series of *n*_*d*_ fluorescence values for each cell. We initially analyze the cells one at a time. We consider the time series for the first cell as a collection of pairs of measurements – $({y}_{0},{y}_{t})$ – with one pair for each positive time point and apply Eq. 6 to each pair. To find E[*Y*_*t*_] and Var[*Y*_*t*_] in Eq. 6, we smooth the time series using a Gaussian process^25^. From the smoothed time series, we estimate E[*Y*_*t*_] for all *t* as the mean of the Gaussian process. Given this mean, we estimate Var[*Y*_*t*_] at each *t* as ${({y}_{t}-{\rm{E}}[{Y}_{t}])}^{2}$. Equation 6 can then be used directly, and we obtain ${n}_{d}-1$ estimates of ${\hat{x}}_{0}$ for that cell. We repeat this process for all cells and therefore obtain ${n}_{r}({n}_{d}-1)$ estimates of ${\hat{x}}_{0}$ in total, which we pool. We take the mode of the resulting distribution as the best estimate for the number of molecules.

### Comparison with biochemical estimates of protein numbers

To verify our method, we compare our results with biochemical measurements of the numbers of molecules. We selected six proteins from budding yeast that have a range of absolute numbers – Fus3, Hog1, Guk1, Def1, Gpm1, and Pgk1 – and subjected GFP-fusions of these proteins^20^ to a photobleaching analysis (Methods). We compare with a unified data set from 19 separate biochemical experiments^26^, involving either mass spectrometry, GFP-tagging and microscopy, or TAP-tagging with immunoblotting.

**Figure 3:**
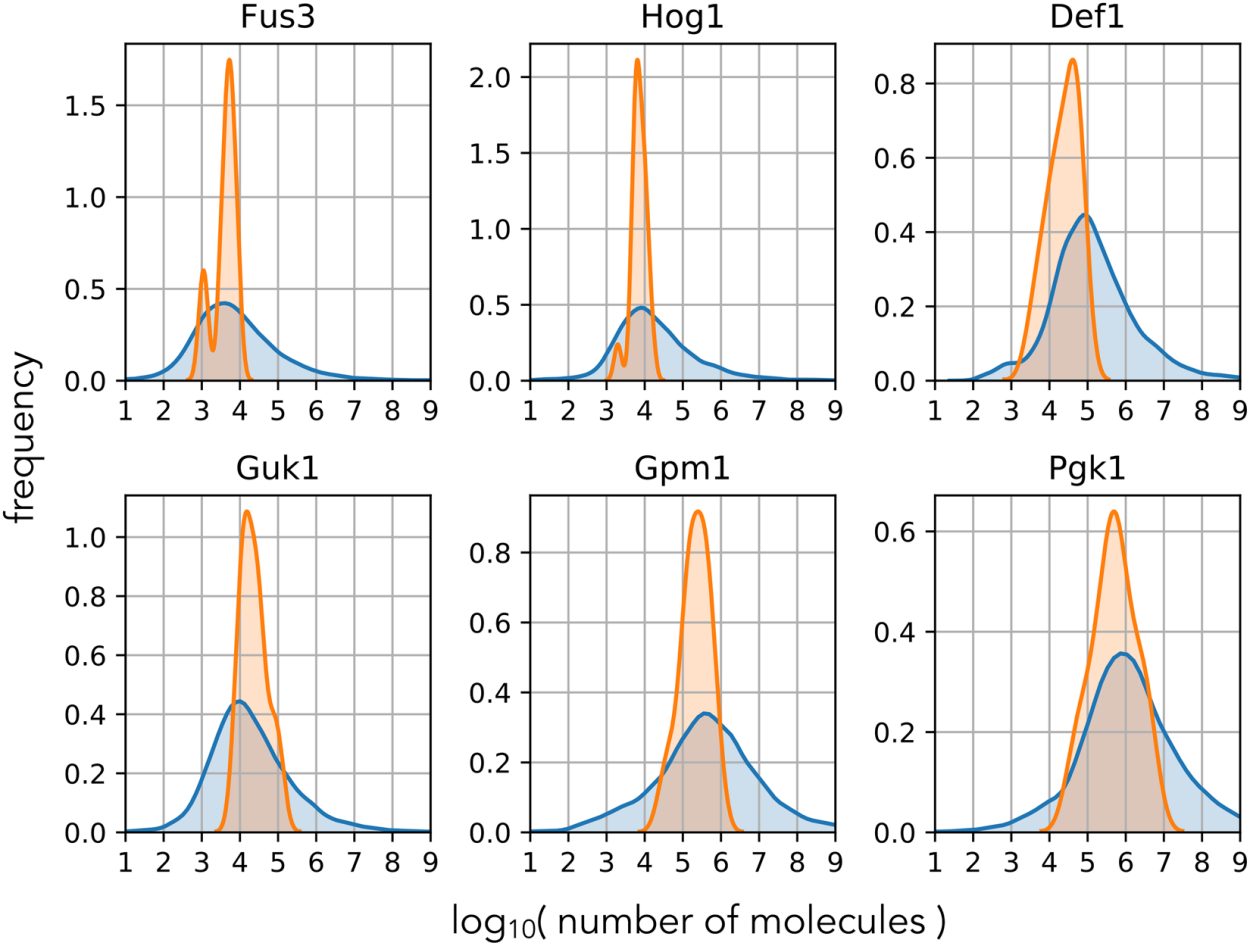
A photobleaching analysis identifies numbers of molecules in agreement with biochemical measurements. We show the distribution comprising the estimates of *x*_0_ for all pairs of *y*_0_ and *y*_*t*_ for $t > 0$ in blue and the distribution of results from 19 biochemical experiments in orange. The numbers of molecules range from approximately 10^3^ (Fus3) to 10^6^ (Pgk1). For all proteins, the mode of the two distributions are within an order of magnitude. Each cell was imaged 45 times in 10 s intervals, and the numbers of cells are as follows: 355 for Fus3; 114 for Hog1; 239 for Def1; 310 for Guk1; 242 for Gpm1; and 311 for Pgk1.

For all proteins, there is an agreement within an order of magnitude between the two approaches (Fig. 3) showing that the measurement noise is not prohibitively large, at least for yeast.

## Discussion

A challenge in quantitative fluorescence microscopy is converting measurements of fluorescence to absolute units. Absolute units enable the pooling of data from different laboratories and are needed both for models to fit single-cell data^4^ and to validate the values of fitted parameters^27^. As such, absolute units facilitate the long-term success of systems and synthetic biology^28,29^.

We have presented a fluctuation analysis to estimate numbers of fluorescent proteins by deliberately photobleaching cells on a wide-field fluorescence microscope. The method provides a straightforward calibration for time-lapse imaging.

Although our estimate is within an order of magnitude of the numbers estimated using biochemical methods, it is often an underestimate (Fig. 3). This behaviour is consistent with a sufficiently large measurement noise (Eq. 11). Nevertheless, underestimation is countered by the estimate of Var[*Y*_*t*_] in Eq. 6. Using smoothing to find E[*Y*_*t*_] and so Var[*Y*_*t*_] as a function of time from a single time series can underestimate Var[*Y*_*t*_] because the smoothed data – the estimate of E[*Y*_*t*_] – will follow a sufficiently large fluctuation whereas the true mean will not. A too small Var[*Y*_*t*_] overestimates the numbers of molecules. In practice, the measurement noise appears sufficiently high to disrupt large fluctuations so that our estimates of E[*Y*_*t*_] do approximate the true mean, but not too high to undermine using Eq. 6.

We might expect an underestimation too because not all tagged proteins fluoresce. Considering the extreme case of a (long) maturation time of the fluorescent protein of 45 minutes and that its degradation is caused by growth with a (short) doubling time of 100 minutes^30^, then we expect the fluorescence at steady-state to be at least $1/45/(1/45+1/100)\simeq 0.7$ of the total amount of protein. This fraction is, however, large enough that the order of magnitude of our estimate is unchanged and lies within the expected error (width of the blue distributions in Fig. 3).

It is surprising that more principled methods, such as Bayesian inference, work poorly (Methods). Although a single exponential decay does not describe photobleaching in yeast (Fig. 2), we know neither how many exponential decays do nor even if exponential decay itself is correct. Further, both the brightness and time scales associated with any exponentials may be the same for all cells in the population or specific to each cell. Compounding the uncertainty in the model of bleaching, there is no consensus on a model for the measurement noise for fluorescence microscopes, which has been described as normal^10^, log-normal^4^, and with a variance that is a function of fluorescence^31^ (and Methods).

Measurements both in single cells and in absolute numbers are necessary to bring discoveries from different laboratories into one predictive framework. Our method of calibrating fluorescence measurements will help enable such a quantitative, single-cell biology.

## Methods

### Selecting proteins to study

When selecting proteins to test, we looked at three whole-proteome datasets of absolute numbers of protein obtained by quantitative Western blot^32^, mass spectrometry^33^, and fluorescence microscopy^34^. Proteins were selected that appeared in all three data sets. To obtain proteins whose levels would be robust to any stresses from our fixing procedure, we used a protein localisation atlas^35^ to select cytoplasmic proteins that showed no significant fold change under starvation and dithiothreitol- and peroxide-induced stress. From these proteins, we selected four giving a broad range of levels: Def1, Guk1, Gpm1, and Pgk1. To these proteins we added Hog1 because of its regular study in our laboratory^36^ and Fus3 because of the availability of a measurement by fluorescence correlation spectroscopy (FCS)^37^. Taking a cytoplasmic concentration of 180 nM for *Saccharomyces cerevisiae* cells and a three times higher concentration for the nucleus^37^ along with cellular and nuclear volumes of 42 *μ*l and 3 *μ*l^30^, we estimate 5,200 molecules of Fus3 per cell from this FCS data.

During the course of our work, however, a comprehensive collection of whole-proteome data sets was published^26^, and this data is the data we use for comparison. Our estimate for Fus3 from the FCS data is within the range of values reported in this data set.

### Cell preparation

Cells from the open-reading-frame GFP collection^20^ were grown overnight in YEPD (2%) media past the diauxic lag, and 0.5 ml of this culture diluted in 5 ml of fresh media. After 5 hours and at an OD of $\simeq$0.5, the cells were fixed^38^.

### Microscopy and image analysis

All experiments were performed on a Nikon Eclipse Ti inverted microscope, controlled with the Perfect Focus System and custom MATLAB scripts (Mathworks) written for Micromanager^39^. We used a 60X 1.2 NA water immersion objective (Nikon), and images were acquired using an Evolve camera (Photometrics) with a 512 × 512 sensor in CCD mode. Cells were adhered to slides using concanavalin A.

To photobleach, the GFP excitation LED was kept at full power for the duration of the experiment with cells imaged for fluorescence for 400 ms every 10 s. We repeated this procedure for multiple, isolated fields of view and for wild-type cells, which do not express GFP.

Given that we imaged fixed cells, cells were selected and segmented at the first time point from a bright-field image and whole-image registration used to propagate this outline to other times. Cells in the bright-field image were chosen by eye to be isolated, well focused, present for the whole experiment (i.e. not washed away), and in a region where the illumination intensity (taken from the flat field correction) was at least 80% of the median illumination. Selected cells were outlined based on the out-of-focus bright-field image using custom MATLAB scripts, and these outlines curated by hand.

Cell fluorescence was calculated as the sum of the values of the brightest 80% of the pixels within the cell boundary. This measure reduced the effect of movements of the stage. We discarded cells that displayed large systematic deviations (such as a sudden drop in fluorescence).

#### Correcting for flat field and background

Fluorescence images were corrected for both flat field and background. We obtained a flat-field image by flowing 0.001% fluorescein (by mass) through a microfluidic device^40^ and imaging multiple positions over a time course. Any microfluidic features were ‘blotted out’ of the images, and the modified images averaged and normalised to a median of one. A correction was applied to the fluorescence images through an element-wise division by the flat field. To remove any background particular to a slide, we subtracted the average pixel fluorescence for a region of the image containing no cells from each pixel value. Fluorescence images were also registered to correct for drift in the field of view.

#### Correcting for autofluorescence

Cells were also corrected for autofluorescence. We corrected bleached wild-type cells for flat field and background, and their average value was subtracted from all fluorescent cells at each time point.

### Inference fails assuming mono-exponential decay

Analysing the data using a previously proposed estimator^14^ and following the procedure of Kim *et al*.^17^, we find that numbers of proteins are underestimated by several orders of magnitude (the maximum number of molecules over all six proteins is approximately 150). This failure is because that method both uses a poor approximation to the mean behaviour of the photobleaching of individual cells (the means in our data are not well described by exponential decay with one time scale) and its sensitivity to the quality of the fit even when the photobleaching is a decay process with a single time scale.

### A maximimum likelihood estimator for *x*_0_

If there is negligible measurement noise, the data comprises *y*_0_, ${\rm{E}}[Y]={\mu }_{y}$, and ${\rm{Var}}[Y]={\sigma }_{y}^{2}$, and the likelihood of *x*_0_ is $P({y}_{0},{\mu }_{y},{\sigma }_{y}^{2}|{x}_{0})$, which we denote ${\mathcal L}$, then we can write explicit expressions.

Using the rules of probability, and writing $\ell ={{\rm{e}}}^{-\lambda t}$ for a particular *λ* and *t*,

$$\begin{array}{rcl} {\mathcal L}  & = & \int \,d\nu \,d{\mu }_{x}\,d{\sigma }_{x}^{2}\,d\ell \,P({y}_{0},{\mu }_{y},{\sigma }_{y}^{2}|\nu ,{\mu }_{x},{\sigma }_{x}^{2},\ell ,{x}_{0})\\  &  & \times \,P(\nu ,{\mu }_{x},{\sigma }_{x}^{2},\ell |{x}_{0})\\  & = & \int \,d\nu \,d{\mu }_{x}\,d{\sigma }_{x}^{2}\,d\ell \,P({y}_{0},{\mu }_{y},{\sigma }_{y}^{2}|\nu ,{\mu }_{x},{\sigma }_{x}^{2},\ell ,{x}_{0})\\  &  & \times \,P({\sigma }_{x}^{2}|\nu ,{\mu }_{x},\ell ,{x}_{0})P(\nu ,{\mu }_{x},\ell |{x}_{0})\\  & = & \int \,d\nu \,d{\mu }_{x}\,d{\sigma }_{x}^{2}\,d\ell \,P({y}_{0}|\nu ,{x}_{0})P({\mu }_{y}|{\mu }_{x},\nu )P({\sigma }_{y}^{2}|{\sigma }_{x}^{2},\nu )\\  &  & \times \,P({\sigma }_{x}^{2}|{\mu }_{x},\ell ,{x}_{0})P({\mu }_{x}|\ell ,{x}_{0})P(\ell )P(\nu \mathrm{).}\end{array}$$

Assuming $P(\ell )$ is uniform between 0 and 1, we can write

15 $$\begin{array}{rcl} {\mathcal L}  & = & \int \,d\nu \,d{\mu }_{x}\,d{\sigma }_{x}^{2}\,d\ell \,\delta ({y}_{0}-\nu {x}_{0})\delta ({\mu }_{y}-\nu {\mu }_{x})\delta ({\sigma }_{y}^{2}-{\nu }^{2}{\sigma }_{x}^{2})\\  &  & \times \,\delta ({\sigma }_{x}^{2}-{\mu }_{x}\mathrm{(1}-\ell ))\delta ({\mu }_{x}-{x}_{0}\ell )P(\nu )\end{array}$$

following Eqs 2 and 3.

We use the identity

16 $$\delta (a-\nu x)=\frac{1}{\nu }\delta (x-\frac{a}{\nu })$$

to evaluate the integrals.

First, we perform the integral over *μ*_*x*_ using the first delta function in *μ*_*x*_

17 $$\begin{array}{rcl} {\mathcal L}  & = & \int \,d\nu \,d{\sigma }_{x}^{2}\,d\ell \frac{1}{\nu }\delta ({y}_{0}-\nu {x}_{0})\delta ({\sigma }_{y}^{2}-{\nu }^{2}{\sigma }_{x}^{2})\\  &  & \times \,\delta ({\sigma }_{x}^{2}-\frac{{\mu }_{y}}{\nu }\mathrm{(1}-\ell ))\delta (\frac{{\mu }_{y}}{\nu }-{x}_{0}\ell )P(\nu )\end{array}$$

and then over ${\sigma }_{x}^{2}$

18 $$ {\mathcal L} =\int \,d\nu \,d\ell \frac{1}{{\nu }^{3}}\delta ({y}_{0}-\nu {x}_{0})\delta (\frac{{\sigma }_{y}^{2}}{{\nu }^{2}}-\frac{{\mu }_{y}}{\nu }(1-\ell ))\delta (\frac{{\mu }_{y}}{\nu }-{x}_{0}\ell )P(\nu ).$$

Integrating over $\ell$, we have

19 $$ {\mathcal L} =\int \,d\nu \frac{1}{{\nu }^{3}{x}_{0}}\delta ({y}_{0}-\nu {x}_{0})\delta (\frac{{\sigma }_{y}^{2}}{{\nu }^{2}}-\frac{{\mu }_{y}}{\nu }(1-\frac{{\mu }_{y}}{\nu {x}_{0}}))P(\nu )$$

and over *ν* gives

20 $$\begin{array}{rcl} {\mathcal L}  & = & \frac{{x}_{0}}{{y}_{0}^{3}}\delta (\frac{{\sigma }_{y}^{2}{x}_{0}^{2}}{{y}_{0}^{2}}-\frac{{\mu }_{y}{x}_{0}}{{y}_{0}}(1-\frac{{\mu }_{y}}{{y}_{0}}))P({y}_{0}/{x}_{0})\\  & = & \frac{1}{{y}_{0}{\sigma }_{y}^{2}}\delta ({x}_{0}-\frac{{\mu }_{y}({y}_{0}-{\mu }_{y})}{{\sigma }_{y}^{2}})P({y}_{0}/{x}_{0})\end{array}$$

where we have brought ${x}_{0}/{y}_{0}^{2}$ into and ${\sigma }_{y}^{2}$ out of the delta function.

For a uniform prior for *ν* (so that $P({y}_{0}/{x}_{0})$ is constant), the likelihood, Eq. 20, is maximized when

21 $${x}_{0}=\frac{{\mu }_{y}({y}_{0}-{\mu }_{y})}{{\sigma }_{y}^{2}}$$

which is Eq. 6.

### Estimating E[Y_t_]

We smooth each cell’s time series, *y*_*t*_, to estimate its mean, ${\rm{E}}[{Y}_{t}]$. To perform the smoothing, we use a Gaussian process with a covariance given by a squared exponential function – $k(x,x^{\prime} )={\theta }_{0}\,\exp [\,-\,{\theta }_{1}{(x-x^{\prime} )}^{2}/2]$ – and determine the hyperparameters for each cell by maximizing the marginal likelihood^25^. These hyperparameters are bounded *a priori*: ${10}^{3} < {\theta }_{0} < {10}^{14}$, ${10}^{-8} < {\theta }_{1} < 1$, and *θ*_2_, which controls the magnitude of the estimated measurement noise, being restricted to $10 < {\theta }_{2} < {10}^{10}$. We use an implementation in Python^41^.

Using simulated data and normally distributed measurement noise, we find that there is an optimum range for the magnitude of the measurement noise. If measurement noise is too low, the estimated mean follows fluctuations in the data and at times is closer to the data than the true mean. Therefore Var[*Y*_*t*_] in Eq. 6 is too small, and the estimate of *x*_0_ is too large. If measurement noise is too high, the estimated mean can sometimes be further from and sometimes closer to the data than the true mean, but the estimator in any case then underestimates (Eq. 9). For intermediate magnitudes of the measurement noise, Eq. 6 is accurate, and the measurement noise prevents fluctuations being long-lived and so corrects for bias in the estimate of E[*Y*_*t*_] and Var[*Y*_*t*_].

The estimator can perform poorly if the time scales of bleaching are shorter than the the duration of the experiment. Then, all the molecules are bleached at late times, and the resulting noisy data can undermine the estimate of E[*Y*_*t*_]. The low numbers of molecules also mean that the linear noise approximation and Eqs 2 and 3 are potentially no longer valid.

### Inference with an explicit model of bleaching and measurement noise

In general, given data ${{\bf{Y}}}_{t}=\{{y}_{1},\ldots ,{y}_{t}\}$, we wish to infer ${\boldsymbol{\theta }}=\{{{\boldsymbol{\theta }}}_{m},{{\boldsymbol{\theta }}}_{e}\}$, where ***θ***_*m*_ are the parameters for the biophysical model of the dynamics of the underlying numbers of proteins, **x**, and ***θ***_*e*_ are the parameters for the distribution of the measurement noise. The *y* variables are related to the **x** variables only through this measurement noise.

The dynamics of the protein numbers, **x**, are determined by chemical reactions and can be described by a master equation for $P({\bf{x}},t)$, the probability distribution of the state **x** at time *t*^19^. We use the linear noise approximation (first-order terms in an expansion of the master equation in the size of the system – the volume of a cell^19^). This approximation makes $P({\bf{x}},t)$ a normal distribution if the initial distribution is either a normal or a delta function.

If we let the stochiometric matrix be **S** and the hazards (propensities) be **h** and noting that **x** describes numbers of molecules not concentrations, then

22 $$P({\bf{x}},t)={\mathscr{N}}({\bf{x}};\mu (t),{\boldsymbol{\Sigma }}(t))$$

where ${\mathscr{N}}$ denotes a normal distribution and *μ*(*t*), its mean, and ${\boldsymbol{\Sigma }}(t)$, its covariance matrix, obey^24^

23 $$\frac{d}{dt}\mu ={{\bf{S}}}^{T}{\bf{h}}(\mu ,t)$$

and

24 $$\frac{d}{dt}{\boldsymbol{\Sigma }}={\bf{J}}{\boldsymbol{\Sigma }}+{\boldsymbol{\Sigma }}{{\bf{J}}}^{T}+{{\bf{S}}}^{T}{\bf{H}}{\bf{S}}$$

with **J** as the Jacobian:

25 $${J}_{ij}=\frac{\partial }{\partial {x}_{j}}\,\sum _{k}\,{S}_{ik}{h}_{k}(\mu ,t)$$

and **H** as a matrix of zeros with ${\bf{h}}(\mu ,t)$ on the diagonal.

We let the biophysical model have two decay processes (Fig. 2). For each cell, indexed by *j*, there are two pools of fluorescence proteins, ${x}_{1,j}$ and ${x}_{2,j}$, which bleach at constant, but cell-dependent, rates, ${\lambda }_{1,j}$ and ${\lambda }_{2,j}$:

26 $${x}_{1,j}\,\mathop{\longrightarrow }\limits^{{\lambda }_{1,j}}\varnothing \,{x}_{2,j}\,\mathop{\longrightarrow }\limits^{{\lambda }_{2,j}}\varnothing $$

Equations 23 and 24 then give

27 $${\mu }_{i,j}={x}_{i,j}^{(0)}{{\rm{e}}}^{-{\lambda }_{i,j}t}$$

and

28 $$\begin{array}{rcl}{\Sigma }_{ii,j} & = & {\Sigma }_{ii,j}^{(0)}{{\rm{e}}}^{-2{\lambda }_{i,j}t}+{{\rm{e}}}^{-{\lambda }_{i,j}t}(1-{{\rm{e}}}^{-{\lambda }_{i,j}t}){x}_{i,j}^{\mathrm{(0)}}\\ {\Sigma }_{12,j} & = & {\Sigma }_{\mathrm{12,}j}^{(0)}{{\rm{e}}}^{-({\lambda }_{\mathrm{1,}j}+{\lambda }_{\mathrm{2,}j})t}\end{array}$$

where initial values have superscripts of zero.

We use a measurement noise that depends on the numbers of fluorescence proteins giving a standard deviation that scales with the mean. If ${y}_{j}(t)$ is the measured fluorescence then

29 $$\begin{array}{rcl}{y}_{j}(t) & \sim  & {\mathscr{N}}({y}_{j}(t);\nu [{x}_{1,j}(t)+{x}_{2,j}(t)]+{f}_{j},\\  &  & {\sigma }_{e,0}^{2}+\nu [{x}_{1,j}(t)+{x}_{2,j}(t)]{\sigma }_{e,1}^{2}+{\nu }^{2}{[{x}_{1,j}(t)+{x}_{2,j}(t)]}^{2}{\sigma }_{e,2}^{2})\end{array}$$

for constant ${\sigma }_{e,i}$ and any residual autofluorescence *f*_*j*_. This model does not have the positive skewness of log-normal noise and has support for negative fluorescence values, which we observe after correcting images for background fluorescence.

### Linear noise and sequential data – a Kalman filter

We wish to infer ***θ*** given **Y**_*t*_. Bayes’s rule states:

30 $$P({\boldsymbol{\theta }}|{{\bf{Y}}}_{t})\propto P({{\bf{Y}}}_{t}|{\boldsymbol{\theta }})P({\boldsymbol{\theta }})$$

or, by using the rules of probability to factorize the likelihood,

31 $$P({\boldsymbol{\theta }}|{{\bf{Y}}}_{t})\propto P({\boldsymbol{\theta }})\,\mathop{\prod }\limits_{i=1}^{t}\,P({y}_{i}|{{\bf{Y}}}_{i-1},{\boldsymbol{\theta }}).$$

We sequentially find each term in Eq. 31 by considering the dynamics of **x** from one time point to the next and then correcting that dynamics given the observed data^24^. Assume that at time point $i-1$ the distribution $P({{\bf{x}}}_{i-1}|{{\bf{Y}}}_{i-1},{\boldsymbol{\theta }})$ is normal with a known mean ${\mu }_{i-1}^{\ast }$ and covariance matrix ${{\boldsymbol{\Sigma }}}_{i-1}^{\ast }$:

32 $$P({{\bf{x}}}_{i-1}|{{\bf{Y}}}_{i-1},{\boldsymbol{\theta }})={\mathscr{N}}({{\bf{x}}}_{i-1};{\mu }_{i-1}^{\ast },{{\boldsymbol{\Sigma }}}_{i-1}^{\ast }).$$

Using the linear noise approximation for the dynamics of **x**, we can, with Eq. 32 providing the initial condition, integrate Eqs 23 and 24 over one time interval to time point *i* to find *μ*_*i*_ and ${{\boldsymbol{\Sigma }}}_{i}$ and that

33 $$P({{\bf{x}}}_{i}|{{\bf{Y}}}_{i-1},{\boldsymbol{\theta }})={\mathscr{N}}({{\bf{x}}}_{i};{\mu }_{i},{{\boldsymbol{\Sigma }}}_{i}).$$

We next wish to extend the conditioning in Eq. 33 to include the data point, *y*_*i*_, at time point *i*. To do so, note that

34 $$\begin{array}{rcl}P({{\bf{x}}}_{i}|{{\bf{Y}}}_{i},{\boldsymbol{\theta }}) & = & P({{\bf{x}}}_{i}|{y}_{i},{{\bf{Y}}}_{i-1},{\boldsymbol{\theta }})\\  & = & \frac{P({y}_{i}|{{\bf{x}}}_{i},{{\bf{Y}}}_{i-1},{\boldsymbol{\theta }})P({{\bf{x}}}_{i}|{{\bf{Y}}}_{i-1},{\boldsymbol{\theta }})}{P({y}_{i}|{{\bf{Y}}}_{i-1},{\boldsymbol{\theta }})}\\  & \propto  & P({y}_{i}|{{\bf{x}}}_{i},{\boldsymbol{\theta }})P({{\bf{x}}}_{i}|{{\bf{Y}}}_{i-1},{\boldsymbol{\theta }})\end{array}$$

using Bayes’s rule, conditioning on ${{\bf{Y}}}_{i-1}$ and ***θ***, and assuming that the measurement noise only depends on the current value of **x**. If the measurement noise too has a normal distribution

35 $$P(y|{\bf{x}},{\boldsymbol{\theta }})={\mathscr{N}}(y;{\bf{U}}{\bf{x}},{\bf{V}})$$

with **U** being a constant projection matrix and **V** being a covariance matrix, then we can simplify Eq. 34 using the properties of normal distributions. We find that $P({{\bf{x}}}_{i}|{{\bf{Y}}}_{i},{\boldsymbol{\theta }})$ is also normal with a mean ${\mu }_{i}^{\ast }$ and a covariance ${{\boldsymbol{\Sigma }}}_{i}^{\ast }$ that satisfy^24^

36 $$\begin{array}{rcl}{\mu }_{i}^{\ast } & = & {\mu }_{i}+{{\boldsymbol{\Sigma }}}_{i}{{\bf{U}}}^{T}{({\bf{U}}{{\boldsymbol{\Sigma }}}_{i}{{\bf{U}}}^{T}+{\bf{V}})}^{-1}({y}_{i}-{\bf{U}}{\mu }_{i})\\ {{\boldsymbol{\Sigma }}}_{i}^{\ast } & = & {{\boldsymbol{\Sigma }}}_{i}+{{\boldsymbol{\Sigma }}}_{i}{{\bf{U}}}^{T}{({\bf{U}}{{\boldsymbol{\Sigma }}}_{i}{{\bf{U}}}^{T}+{\bf{V}})}^{-1}(\,-\,{\bf{U}}{{\boldsymbol{\Sigma }}}_{i}).\end{array}$$

The predictions of *μ*_*i*_ and ${{\boldsymbol{\Sigma }}}_{i}$ found from ${\mu }_{i-1}^{\ast }$ and ${{\boldsymbol{\Sigma }}}_{i-1}^{\ast }$ using the linear noise approximation are corrected to ${\mu }_{i}^{\ast }$ and ${{\boldsymbol{\Sigma }}}_{i}^{\ast }$ because of the new data point and the measurement noise.

The factors in Eq. 31, $P({y}_{i}|{{\bf{Y}}}_{i-1},{\boldsymbol{\theta }})$ obey

37 $$P({y}_{i}|{{\bf{Y}}}_{i-1},{\boldsymbol{\theta }})=\int \,d{{\bf{x}}}_{i}P({y}_{i}|{{\bf{x}}}_{i},{\boldsymbol{\theta }})P({{\bf{x}}}_{i}|{{\bf{Y}}}_{i-1},{\boldsymbol{\theta }})$$

and are therefore the normalizing factors for Eq. 34, satisfying

38 $$P({y}_{i}|{{\bf{Y}}}_{i-1},{\rm{\theta }})={\mathscr{N}}({y}_{i};{\bf{U}}{{\boldsymbol{\mu }}}_{i},{\bf{U}}{{\boldsymbol{\Sigma }}}_{i}{{\bf{U}}}^{T}+{\bf{V}}\mathrm{).}$$

Hence from a normal prior distribution for **x**_1_, $P({{\bf{x}}}_{1}|{\boldsymbol{\theta }})$, we use Eq. 38 to find $P({y}_{1}|{\boldsymbol{\theta }})$, the first term in the factorization of the likelihood (Eq. 31) and Eq. 36 to find $P({{\bf{x}}}_{1}|{y}_{1},{\boldsymbol{\theta }})$, the starting normal distribution in Eq. 32 for the sequential inference.

To specialize the algorithm to photobleaching, the matrices in Eq. 35 are ${\bf{U}}=[\nu \,\nu ]$ and ${\bf{V}}={\sigma }_{e}^{2}$, a constant. We subtract the autofluorescence, *f*_*j*_, from each data point before applying the Kalman filter. The Kalman update, Eq. 36, can result in unphysical, negative components of *μ*, which we set to zero.

We must specify a prior $P({{\bf{x}}}_{1}|{{\boldsymbol{\theta }}}_{m})$ to begin the inference scheme. To do so, we introduce two parameters: *x*_0_, which is the total amount of fluorescent protein at $t=0$, and *α*, which is the partitioning of this fluorescent protein between the two pools. We infer both these parameters. For the Kalman filter, we require that $P({{\bf{x}}}_{1}|{{\boldsymbol{\theta }}}_{m})$ be a normal distribution, but we wish to start **x**_1_ at a known value and so use

39 $${\mu }_{1}^{\ast }={x}_{0}[\begin{array}{c}1-\alpha \\ \alpha \end{array}]\,{\rm{and}}\,{\Sigma }_{1}^{\ast }=[\begin{array}{cc}0 & 0\\ 0 & 0\end{array}].$$

so that the normal distribution approximates a delta function.

### Extending to state-dependent measurement noise

The variance of the measurement noise of Eq. 29 depends on **x**, but our inference scheme assumes a constant variance (Eq. 35). We therefore approximate Eq. 29 by replacing the explicit dependence of the variance on **x** by its expected value given ${{\bf{Y}}}_{j-1}$:

40 $$\begin{array}{ccc}{y}_{j}|{{\bf{x}}}_{j} & \sim  & {\mathscr{N}}({y}_{j};{\bf{U}}{x}_{j}+{f}_{j},{\sigma }_{e,0}^{2}+{\bf{U}}{{\bf{x}}}_{j}{\sigma }_{e,1}^{2}+{({\bf{U}}{{\bf{x}}}_{j}{\sigma }_{e,2})}^{2})\\  & \simeq  & {\mathscr{N}}({y}_{j};{\bf{U}}{{\bf{x}}}_{j}+{f}_{j},{{\rm{E}}}_{{{\bf{x}}}_{j}|{{\bf{Y}}}_{j-1}}[{\sigma }_{e,0}^{2}+{\bf{U}}{{\bf{x}}}_{j}{\sigma }_{e,1}^{2}+{({\bf{U}}{{\bf{x}}}_{j}{\sigma }_{e,2})}^{2}])\\  & = & {\mathscr{N}}({y}_{j};{\bf{U}}{{\bf{x}}}_{j}+{f}_{j},{\sigma }_{e,0}^{2}+{\bf{U}}{\mu }_{j}{\sigma }_{e,1}^{2}+{\bf{U}}({\mu }_{j}{\mu }_{j}^{{\rm{T}}}+{{\boldsymbol{\Sigma }}}_{j}){{\bf{U}}}^{{\rm{T}}}{\sigma }_{e,2}^{2})\end{array}$$

The usual update in the Kalman filter, Eq. 36, can then be used.

### Sampling the posterior probability

To sample from $P({\boldsymbol{\theta }}|{{\bf{Y}}}_{t})$ in Eq. 30 we use both optimization and a Markov chain Monte Carlo method.

We distinguish between heterogenous parameters, which are specific to each cell (*λ*_1_, *λ*_2_, *f*, *x*_0_ and *α*), and homogeneous parameters, which have the same values for all cells (the *σ*_*e*,*i*_ and *ν*). The homogenous parameters and *x*_0_ have scale-free priors: for example, $P(\nu )=1/\nu$. The heterogenous parameters other than *x*_0_ have flat priors. All priors are proper and bounded to physical values. To ensure the expected behaviour is dependent only on the heterogeneous parameters and to improve the mixing of the Markov chain, we propose the combination *νx*_0_, referred to as *y*_0_, rather than *x*_0_.

To generate samples from the posterior, we use a Metropolis-within-Gibbs scheme^42^ with the heterogeneous parameters updated separately from the homogeneous parameters. We employ adaptive parallel tempering to accelerate mixing in the Gibbs sampler^43^, which performs well on benchmark biochemical models^44^. Specifically, we use 10 chains with their temperatures chosen adaptively. Parameters are proposed independently, with *λ*_1_, *λ*_2_, *f* and *α* proposed from normal distributions and *y*_0_, the ${\sigma }_{{e}_{i}}$, and *ν* proposed from log-normal distributions. We adaptively select the scales for the proposal distributions for the heterogeneous and homogeneous parameters.

To start the Monte Carlo method, we try to find parameters that maximize the likelihood $P({{\bf{Y}}}_{t}|{\boldsymbol{\theta }})$. We use a nested optimisation scheme^45^:

1. We find starting values for the heterogeneous parameters by fitting a bi-exponential decay to each cell’s time series.
2. All parameters, including the heterogenous parameters, are then fitted for each cell, independently of all other cells, using a particle swarm.
3. We create an initial parameter set for the homogenous parameters by taking the median of the homogeneous parameters from the individual fits of Step 2.
4. We perform iterative optimisation: first optimising the homogeneous parameters with the heterogeneous parameters fixed then vice versa until a maximum is reached. Each individual optimisation uses Matlab’s fmincon.
5. To provide diverse starting points for the chains, half of our chains are initialised at the parameter values found in Step 4 and the other half are initialised from optima found by performing the iterative optimisation of Step 4 from random parameters rather than from those found in Step 3.

### Estimating the expected numbers of proteins

Given a sample of parameters from the posterior distribution $P({\boldsymbol{\theta }}|{\bf{Y}})$, and a fluorescence measurement *y*, we would like to find the posterior distribution of the number of proteins, *x*:

41 $$P(x|y,{\bf{Y}})=\int \,d{\boldsymbol{\theta }}P(x|y,{\boldsymbol{\theta }})P({\boldsymbol{\theta }}|y,{\bf{Y}})P(y).$$

We assume that the measurement *y* does not change the posterior probability of ***θ*** (because there is substantially more data in **Y**): $P({\boldsymbol{\theta }}|y,{\bf{Y}})\simeq P({\boldsymbol{\theta }}|{\bf{Y}})$. Ignoring *P*(*y*), which is independent of *x*, we have

42 $$P(x|y,{\bf{Y}})\propto \int \,d{\boldsymbol{\theta }}P(x|y,{\boldsymbol{\theta }})P({\boldsymbol{\theta }}|{\bf{Y}}).$$

By Bayes’s rule

43 $$\begin{array}{lll}P(x|y,{\boldsymbol{\theta }}) & \propto  & P(y|x,{\boldsymbol{\theta }})P(x)\\  & \propto  & P(y|x,{\boldsymbol{\theta }})\end{array}$$

assuming a constant prior, *P*(*x*). We approximate *νx* by *y* in Eq. 29 so that

44 $$\begin{array}{lll}P(x|y,{\boldsymbol{\theta }}) & \mathop{\propto }\limits_{ \tilde {}} & {\mathscr{N}}(y;y+f,{\sigma }_{e,0}^{2}+y{\sigma }_{e,1}^{2}+{(y{\sigma }_{e,2})}^{2})\\  & = & {\mathscr{N}}(x;\frac{y-f}{\nu },\frac{{\sigma }_{e,0}^{2}+y{\sigma }_{e,1}^{2}+{(y{\sigma }_{e,2})}^{2}}{{\nu }^{2}})\end{array}$$

normalizing over *x* and using the properties of normal distributions. We use Eq. 44 to evaluate Eq. 42 as an average over the Monte Carlo samples generated from $P({\boldsymbol{\theta }}|{\bf{Y}})$ on a grid of *x* values.

To extent these results to measurements of fluorescence over a population of cells where we are interested in the distribution of $\bar{x}=\sum \,{x}_{i}/N$, we can use that the distribution of a sum of independent normal variables is also normal with a mean equal to the sum of the means of the variables in the sum and with a variance equal to the sum of the variances^19^. Equation 44 then becomes

45 $$\begin{array}{ccc}P(x|{\bf{y}}{\boldsymbol{,}}{\boldsymbol{\theta }}{\boldsymbol{)}} & = & {\mathscr{N}}(\bar{x};\frac{\bar{y}-\bar{f}}{\nu },\frac{{\sigma }_{e,0}^{2}+\bar{y}{\sigma }_{e,1}^{2}+\bar{{y}^{2}}{{\sigma }_{e,2}}^{2}}{N{\nu }^{2}})\\  & \simeq  & \delta (\bar{x}-\frac{\bar{y}}{\nu })\end{array}$$

if ${\sigma }_{e,i}^{2}/N$ and $\bar{f}/\bar{y}$ are sufficiently small. Using a kernel density representation for $P(\bar{x}|{\bf{y}},{\bf{Y}})$, with kernel $K(x,x^{\prime} )=\,\log \,{\mathscr{N}}(x;x^{\prime} ,{\sigma }_{K})$ and ${\sigma }_{K}^{2}={10}^{-2}$, we can write that

46 $$\begin{array}{rcl}P(x|{\bf{y}},{\bf{Y}}) & = & \int \,dx^{\prime} \,K(\bar{x},x^{\prime} )P(x^{\prime} |{\bf{y}},{\bf{Y}})\\  & \simeq  & \int \,d\nu dx^{\prime} \,K(\bar{x},x^{\prime} )\delta (x^{\prime} -\frac{\bar{y}}{\nu })P(\nu |{\bf{Y}})\\  & = & \int \,d\nu \,K(\bar{x},\frac{\bar{y}}{\nu })P(\nu |{\bf{Y}})\end{array}$$

using Eqs 42 and 45. We evaluate the integral in Eq. 46 using the Monte Carlo samples of $P(\nu |{\bf{Y}})$.

### Combining estimates from different experimental replicates

Writing $\{{\mathscr{D}}\}=\{{{\mathscr{D}}}_{1},{{\mathscr{D}}}_{2}\ldots {{\mathscr{D}}}_{N}\}$ as the set of data from all replicates, we wish to sample from $P({\boldsymbol{\theta }}|\{{\mathscr{D}}\})$. We note that the datasets are conditionally independent given ***θ*** so that

47 $$\begin{array}{lll}P({\boldsymbol{\theta }}|\{{\mathscr{D}}\}) & \propto  & P({\boldsymbol{\theta }})\,\mathop{\prod }\limits_{i}^{N}\,P({{\mathscr{D}}}_{i}|{\boldsymbol{\theta }})\\  & \propto  & P({\boldsymbol{\theta }})\,\prod ^{N}\,\frac{P({\boldsymbol{\theta }}|{{\mathscr{D}}}_{i})}{P({\boldsymbol{\theta }})}\\  & = & \frac{{\prod }_{i}\,P({\boldsymbol{\theta }}|{{\mathscr{D}}}_{i})}{P{({\boldsymbol{\theta }})}^{N-1}}.\end{array}$$

We approximate the $P({\boldsymbol{\theta }}|{{\mathscr{D}}}_{i})$ in Eq. 47 with kernel density estimates. If $\{{{\boldsymbol{\theta }}}_{i\mathrm{,1}},\ldots {{\boldsymbol{\theta }}}_{i,M}\}$ is the set of parameter samples from the posterior $P({\boldsymbol{\theta }}|{{\mathscr{D}}}_{i})$ obtained from our Monte Carlo method, then

48 $$P({\boldsymbol{\theta }}|{{\mathscr{D}}}_{i})\simeq \frac{1}{M}\,\mathop{\sum }\limits_{j=1}^{M}\,K({\boldsymbol{\theta }},{{\boldsymbol{\theta }}}_{i,j})$$

where the kernel functions are isotropic log normal distributions with variance ${\sigma }_{K}^{2}$ as before. In principle, these kernel density estimates allow Eq. 47 to be evaluated at any ***θ***; in practice, we use a Metropolis algorithm to sample ***θ*** to overcome the dimensionality of the parameter space.

## Results

**Figure 4:**
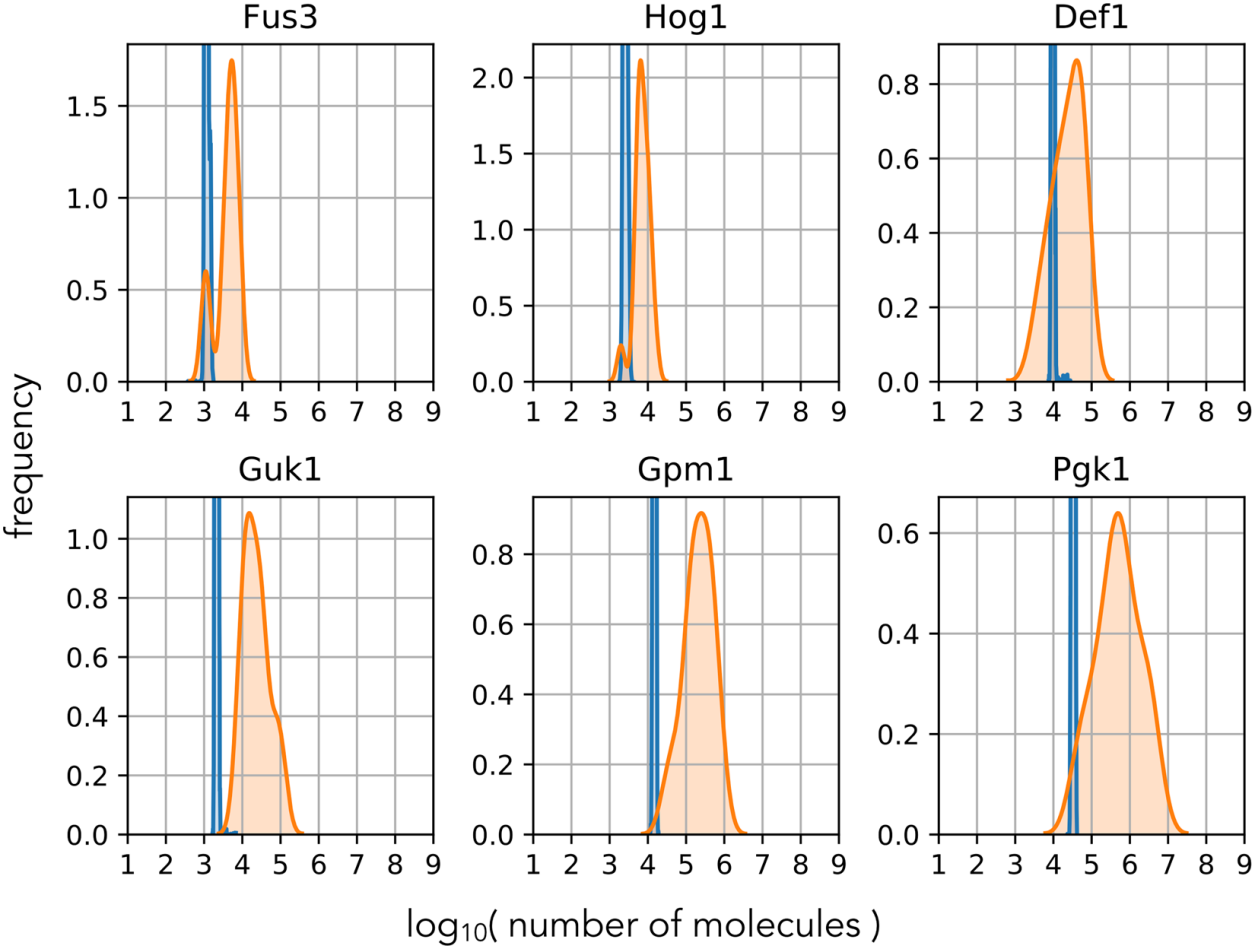
Inference with two heterogeneous decay processes and measurement noise systematically underestimates the numbers of molecules. We show the distribution of protein numbers estimated from the data and our inference of *ν* in blue and the distribution of results from 19 biochemical experiments in orange.

Our results give a lower bound on the number of molecules (Fig. 4). Note that here we ignore the first five time points of each time series because these points can have unexpectedly large fluctuations.

### Modelling measurement noise

To determine the importance of the different contributions to Eq. 29, we used an informative log-normal prior for the parameter *ν* with its mode equal to the median of the value of *ν* estimated from the biochemical data on numbers and its empirical standard deviation equal to half of their interquartile range. Repeating the inference with this prior, we find that $\nu =19.2$ (interquartile range: 19.0 to 19.5), ${\sigma }_{e,0}=136.6$ (interquartile range: 135.1 to 138.5), ${\sigma }_{e,1}=1.31$ (interquartile range: 1.29 to 1.32), and ${\sigma }_{e,2}=0.0027$ (interquartile range: 0.0027 to 0.0028).

These results imply that the term proportional to the number of molecules in the variance of the measurement noise dominates the constant term. For all the proteins studied, the linear term (proportional to ${\sigma }_{e\mathrm{,1}}$ in Eq. 29) is at least an order of magnitude larger that the constant term (${\sigma }_{e\mathrm{,0}}$), and the quadratic term (proportional to ${\sigma }_{e\mathrm{,2}}$) dominates for proteins with high numbers (Gpm1 and Pgk1).

## Data Availability

Data generated in this work is available at 10.7488/ds/2594.
